# Impact of Passive Solar Drying and Storage on Secondary Plant Metabolites and Nitrate Contents of Abyssinian Mustard, Amaranth, and Pumpkin Leaves

**DOI:** 10.1111/1750-3841.70800

**Published:** 2026-01-07

**Authors:** James S. Chacha, Nadja Förster, Susanne Huyskens‐Keil, Christian Ulrichs, Constance Rybak

**Affiliations:** ^1^ Thaer‐Institute of Agricultural and Horticultural Sciences, Division Urban Plant Ecophysiology Humboldt‐Universität zu Berlin Berlin Germany; ^2^ Department of Food Science and Agroprocessing, School of Engineering and Technology Sokoine University of Agriculture Morogoro Tanzania

**Keywords:** African indigenous vegetables, bioactive compounds, drying, health

## Abstract

The present study compared the effect of passive direct solar dryer (PDSD) and passive indirect solar dryer (PISD), each followed by a 30‐day storage period, on the secondary plant metabolite and nitrate contents of amaranth, Abyssinian mustard, and pumpkin leaves. Total carotenoid contents were retained in amaranth (0.70–0.85 mg g^−1^ dry weight [DW]) and pumpkin leaves (1.04–1.16 mg g^−1^ DW) following PDSD and PISD. However, for Abyssinian mustard, a significant decrease of the total carotenoid contents (45% PDSD, 33% PISD) was observed compared to the control (oven‐dried leaves) (0.98 mg g^−1^ DW). Total flavonoid contents were maintained across the vegetables (2.83–78.20 mg g^−1^ DW) irrespective of the drying treatment, except for amaranth leaves, where a significant decline (54% PISD) was observed compared to the control (5.83 mg g^−1^ DW). Although no significant difference was found in total phenolic acid contents between the control and the variants in amaranth, a significantly higher content was observed in pumpkin leaves (25.12 mg g^−1^ DW, PDSD) compared to the control (17.38 mg g^−1^ DW). Exceptionally for pumpkin leaves, a significant decline of the total chlorophyll contents (18%, PDSD; 16%, PISD) was observed after a 30‐day storage compared to the initial contents (8.37 mg g^−1^ DW, PDSD; 6.49 mg g^−1^ DW, PISD). The nitrate contents obtained in this study (80–460 mg kg^−1^ fresh weight [FW], PDSD; 364–915 mg kg^−1^ FW, PISD) were below the maximum allowable limits by the European Commission Regulation (2000–4500 mg kg^−1^ FW), implying the safety of the dried vegetables with respect to nitrate levels.

**Practical Applications:**

This study suggests the utilization of passive solar dryers as a cost‐effective drying alternative for smallholder farmers and households in vegetable quality preservation and minimization of postharvest losses.

## 1 Introduction

Postharvest losses (PHLs) remain a significant challenge in handling agricultural produce due to their link to high food loss and waste, especially in low‐ and middle‐income countries (LMICs) (Gustavsson et al. 2011; Pias et al. 2020). In the LMICs, PHLs in vegetables are associated with inadequacies in postharvest handling, processing, infrastructure, and storage (Karim and Hawlader 2005; Mezquita et al. 2020). For example, in East Africa, losses of at least 40% have been estimated for vegetables and fruits (Ssemwanga et al. 2020). Due to limitations linked to PHLs, the potential benefits obtained from the production, consumption, and marketing of African indigenous vegetables (AIVs) have not been achieved (Francisca and Eyzaguirre 2007; Maseko et al. 2019). First, AIVs face high perishability due to their high metabolic activity and inherent moisture content (Skåra et al. 2023). Second, AIVs are seasonally available as a result of the unavailability or scarcity of suitable processing and storage technologies (Hasan et al. 2019). Consequently, AIVs are highly exposed to spoilage (Noori et al. 2021) and to limited storability and shelf life (Goel et al. 2025).

Despite the challenges linked to PHLs, the promotion of AIVs has received increasing attention due to their diverse composition of secondary plant metabolites with potential health benefits (Neugart et al. 2017; Edith et al. 2018). On the basis of their free radical scavenging, reducing, and metal‐chelating capacities (Thamkaew et al. 2021; Chaves et al. 2022), secondary plant metabolites are potential in the fight against cardiovascular diseases (Ali et al. 2023; Bayang et al. 2024). The secondary plant metabolite contents of vegetables depend on the vegetables’ species and cultivar and on preharvest factors such as soil and climate conditions and handling during harvest, as well as on postharvest factors, mainly the processing and storage conditions (Tiwari and Cummins 2013). An equally important quality indicator of AIVs is the nitrate content, which is highly accumulated in leaves compared to other plant parts as a result of excessive absorption by the plant due to fertilization (Kundu et al. 2024). Thus, nitrate intake in humans could occur through leafy vegetable consumption in the form of dietary nitrate. However, it has been stated that nitrate intake could result in either beneficial effects, for example, improvement of cardiovascular health (Lundberg et al. 2011; Matteini et al. 2025) and the oral microbiome (du Toit et al. 2024); or harmful health effects, for example, cancers and child methemoglobinemia (Colla et al. 2018; Hosseini et al. 2023). Thus, to control the intake of dietary nitrate, accurate knowledge and understanding of the nitrate metabolism of leafy vegetables and possible impacts is significant (Hasheminasab et al. 2025).

One key approach to reduce PHLs in leafy vegetables is to reduce the moisture content through the production of dried products (Hasan et al. 2019). Reducing moisture content to safe limits is necessary for the minimization of undesired inherent biochemical changes that could adversely affect the quality parameters due to deterioration and spoilage (Peñas et al. 2011). The quality of dried agricultural products, including AIVs, depends on the drying process parameters, mainly temperature, relative humidity, and drying duration (Getahun et al. 2021), which could influence the secondary plant metabolite contents in different ways. For example, in a study by Amin et al. (2024), the total carotenoid, chlorophyll, flavonoid, and phenolic acid contents were significantly affected by high relative humidity, low temperature, or by the combination of these factors. According to Pant et al. (2021), abiotic factors (e.g., temperature, light, and UV‐B radiation) exert structural and anatomical modifications on plants, resulting in changes in their chemical components. For shelf life improvement of perishable and temperature‐sensitive foods such as vegetables, drying temperature conditions of between 50°C and 60°C have been recommended (Leon et al. 2002; Owureku‐Asare et al. 2022). However, specific optimum drying conditions may vary depending on the vegetable species with respective moisture contents. In general, for dried products, final moisture contents of 5%–8% have been stated as optimum for the achievement of prolonged storage and preservation (Hande et al. 2014; Salve and Fulambarkar 2021).

For smallholder rural households in the LMICs with limited access to stable electricity, and where alternative sources are still expensive, simple and cost‐effective drying technologies such as passive solar dryers are crucial (Saikia et al. 2022). Passive solar dryers have minimum energy requirements (Srivastava and Shukla 2017; Vijayan et al. 2017) and result in a minimal carbon footprint (Saleh and Badran 2009; Saikia et al. 2022) compared to conventional dryers. Further, compared to open sun drying, passive solar dryers are associated with enhanced product quality obtained through controlled drying of agricultural produce (Salve and Fulambarkar 2021). However, there are limited empirical studies on the effect of passive solar drying on the quality of AIVs, specifically on the secondary plant metabolite and nitrate contents. Mongi and Ngoma (2022) investigated the effect of cabinet and tunnel solar dryers, but with a focus on different mango varieties and only on the nutritional contents (e.g., protein, minerals). Few studies on the secondary plant metabolite and nitrate contents of AIVs have only focused on the combined effect of solar drying and pre‐treatments. A study by Muchoki et al. (2010) showed that solar drying combined with different pre‐treatments (fermentation, acidification, and hot‐water blanching) significantly reduced the nitrate content compared to values obtained in fresh cowpea leaves. Further, a 3‐month storage in polyethylene bags at 18–32°C of the fermented‐solar‐dried samples resulted in significantly lower nitrate contents compared to other samples (fresh and acidified‐solar‐dried) (Muchoki et al. 2010). In a study by Cheptoo et al. (2019), the phenolic acid and flavonoid contents of amaranth and African nightshade were better retained using freeze‐drying than solar drying, with blanching linked to a reduction of the secondary plant metabolites. Thus, the inclusion of pre‐treatments has not always achieved the intended objective of quality preservation in dried vegetables (Oliveira et al. 2016; Reis et al. 2022). Besides, performing pre‐treatments at the household level prior to drying leafy vegetables is impractical, laborious, and time‐consuming.

The aim of the current study was therefore to determine the effect of passive direct and passive indirect solar drying, followed by a 30‐day storage period, on the selected secondary plant metabolite and nitrate contents of amaranth, Abyssinian mustard, and pumpkin leaves. This is to evaluate the available drying techniques for improved shelf life and storability of temperature‐sensitive compounds in AIVs. The three AIVs, namely, amaranth (*Amaranthus hybridus*), pumpkin leaves (*Cucurbita maxima*), and Abyssinian mustard (*Brassica* *carinata*), were selected on the basis of their high consumer preference and fast‐growing capabilities compared to other vegetables in the study sites.

## 2 Materials and Methods

### 2.1 Vegetable Growing and Plant Material

The present study was conducted in the Mkuranga district in Tanzania, located at latitude 7°7′0″ S and longitude 39°12′0″ E of the Equator. The district experiences a modified type of equatorial climate characterized by hot and humid conditions throughout the year, with an average temperature of 28–30°C and an annual rainfall of 800–1100 mm (Mbwambo and Liwenga 2020). Seeds of the three vegetables (amaranth, pumpkin, and Abyssinian mustard) procured from agricultural shops in Dar es Salaam were grown under field conditions using flat gardens near the Nutrition Upscaling Centre (NUC) in the Mkuranga district from June to July 2024. For the production of each AIV, a plot size of 14 × 10 m^2^ was used to grow at least 65 plants per species. Farmyard manure (90 kg plot^−1^) was applied prior to sowing. Irrigation was conducted two times per day (in the morning and evening), at an interval of 2 days until harvest. Six weeks after sowing, the vegetables were harvested from the respective plots by plucking between the leaf stalks and the main stems to obtain a mixed sample. Prior to drying the vegetables, leaf stalks were removed to obtain at least 250 g of whole leaves only for respective drying treatments. Pre‐treatments (e.g., wet cleaning) were not conducted prior to drying.

### 2.2 Experimental Setting and Drying Treatments

**FIGURE 1:**
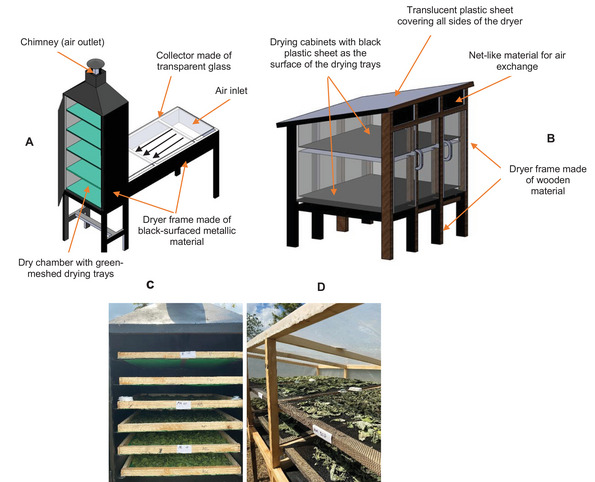
Schematic diagrams of passive indirect solar dryer (A) and passive direct solar dryer (B). Vegetable plant materials in the solar dryers during the drying period (C and D). *Source*: James Chacha.

After harvest, the actual drying experiment was carried out at the NUC, where the passive direct solar dryer (PDSD) and passive indirect solar dryer (PISD) were installed. One PDSD (capacity: 10–12 kg) and one PISD (capacity: 5 kg), designed by Small Industries Development Organization (SIDO) Tanzania (https://www.sido.go.tz/en) and tested in the Vegi‐Leg project (grant number 2816PROC09), were used for drying purposes (Figure 1). Although both PDSD and PISD operate on the basis of the natural buoyant flow of hot air for drying the products (Rezaei et al. 2021), the key difference between the two passive solar dryers lies in their drying mechanism. For PISD, the food product is indirectly hit by solar radiation directed onto the solar collector, and the collected hot air is transferred into the drying chamber, where the food product is placed on shelves (Figure 1A). On the other hand, the drying mechanism for PDSD involves placing the food product on trays inside the enclosure covered with a translucent plastic sheet through which solar radiation directly hits the product (Figure 1B).

In brief, leaf material of the three AIV species was dried via PDSD and PISD for a period of 3 and 4 days, respectively. For both solar dryers, drying was conducted daily from 9 a.m. to 5 p.m. until complete dryness (3 days for PDSD, 4 days for PISD). Drying of the AIVs was conducted only at daytime based on the normal drying procedure of food products at the household level in the study area. Throughout the drying duration, the solar dryers were brought outside the NUC in the mornings (9 a.m.) and returned inside every evening (5 p.m.). The vegetable materials were kept inside the dryers throughout the drying duration, with the doors opened only to check that the data loggers were continuously functioning throughout the drying period. To achieve maximum absorption of solar radiation deemed sufficient for the drying of the AIVs for each day, the solar dryers were positioned in a direction facing the sun. Drying was considered complete based on the brittleness of the dried leaves (Mulokozi and Svanberg 2003) and texture. On the final days of drying using each solar dryer, samples were packed and sealed in 20 mg transparent and airtight plastic containers and stored at room temperature (22–25°C) away from light until further respective compound analyses. Leaf samples ovendried at 40°C (Genlab oven, hot air drying) were used as a control based on a recommendation by ElGamal et al. (2023). For each vegetable species, three experimental variant replications were conducted, each with 250 g of fresh vegetable material. Table 1 provides a detailed description of the different treatments.

**TABLE 1:** Experiment design and sampling.

Variant	Description
Control	Oven drying at 40°C for 1 day
Passive direct solar dryer (PDSD)	Passive direct solar drying for 3 days
Passive indirect solar dryer (PISD)	Passive indirect solar drying for 4 days
30‐day storage of passive direct solardried samples (PDSD–S)	After 30‐day storage of PDSD samples in 20 mg transparent and airtight plastic containers (22–25°C, away from light)
30‐day storage of passive indirect solardried samples (PISD–S)	After 30‐day storage of PISD samples in 20 mg transparent and airtight plastic containers (22–25°C, away from light)

### 2.3 Determination of Drying Conditions

During the drying of the vegetables, the ambient and drying temperatures (°C) and relative humidity (%) of the inlet and outlet air of the solar dryers were recorded using a built‐in sensor, HOBO MX2301 Bluetooth Temperature/RH Data Loggers (Onset‐MX2301A, Germany). Solar light intensity (W m^−2^) was recorded using the HOBO Pendant MX Water Temperature and Light Data Logger (Onset‐MX2202, Germany). All the recorded data were reported on 1‐h intervals of the drying process.

### 2.4 Determination of Moisture Content

Moisture content was determined by the standard ovendrying method (48 h, 105°C). Two grams of vegetable material were obtained from each vegetable species with respect to the experimental treatment variants (control/ovendried, PDSD, and PISD samples). Mean results obtained from triplicate samples were expressed in percentage (%). Moisture content reduction was then determined on the basis of the values obtained before and after solar drying.

### 2.5 Determination of Secondary Plant Metabolite Contents

Carotenoid and chlorophyll contents were determined on the basis of the method described in detail by Edelenbos et al. (2001) and Mageney et al. (2016) with some modifications. Powdered plant materials of 10 mg were extracted with 500 µL methanol–tetrahydrofuran solution (1:1, v: v), followed by drying of the combined supernatants in a nitrogen stream and lastly dilution of the dried pellets with 400 µL methyl *tert*‐butyl‐ether and methanol (2:3). Analysis of extracts was performed using high‐performance liquid chromatography (HPLC) (Ultimate 3000, Thermo Scientific, Germany with an autosampler WPS‐3000TR, pump LPG 3400RS, column compartment TCC‐3000RS, and diode array detector DAD‐3000RS). The Ultimate C18 column (Acclaim 120, C18, 5 µm, 120 Å, 2.1 × 250 mm^2^) was used for separation. Measurements were conducted at 440 nm using two eluents (Eluent A: 80% methanol, Eluent B: ethyl acetate) at the following conditions: flow rate: 0.4 mL min^−1^; oven temperature: 30°C; isocratic run: 00 min 20% B, 2.5 min 22.5% B, 20 min 50% B, 22.5 min 50% B, 24 min 80% B, 26 min 80% B, 31 min 100% B, 34 min 100% B, 42 min 20% B, 47 min 20% B; volume of extract injected: 10 µL. Quantification and identification were done on the basis of commercially available standards and respective calibration curves. The chlorophyll and carotenoid contents were presented in mg g^−1^ dry weight [DW] (*n* = 3).

For the determination of the flavonoids and phenolic acids content, the extraction and HPLC protocol described by Förster et al. (2023) was used. Pulverized plant materials of 20 mg (*n* = 3 for each plant treatment) were extracted with 300 µL of 70% MeOH (v/v; pH = 4, acetic acid) and 100 µL of internal standard (4‐methoxycinnamic acid, 1 mM, Sigma Aldrich, Germany). Subsequently, sonification was performed in an ice bath (Bandelin SONOREX, BANDELIN electronic GmbH & Co. KG, Germany), and finally vacuum concentration with no temperature to near dryness (vacuum concentrator, Thermo Scientific Savant SPD111V Concentrator, vacuum pump: Vacuumbrand PC 3001 series, CVC3000, Germany). Quantification of the extracts was performed using HPLC (Ultimate 3000, Thermo Scientific, Germany with an autosampler WPS‐3000TR, pump LPG‐3400RS, column compartment TCC‐3000RS, and diode array detector DAD‐3000RS), whereas the C16 column (Acclaim PA, 3 µm, 150 × 2.1 mm^2^, Thermo Scientific, Germany) protected by a pre‐column (5 µm, 120 Å, 2 × 10 mm^2^, Thermo Scientific) was used for separation. Measurements were performed at 290 nm using two analytical solvents (A: H_2_O [0.5% formic acid], B: 40% acetonitrile). The following conditions were observed: flow rate: 0.4 m min^−1^; oven temperature: 35°C; isocratic run: 0–1 min: 0.5% B, 1–10 min: 0.5%–40% B, 10–12 min: 40% B, 12–18 min: 40%–80% B, 18–20 min: 80% B, 20–24 min: 80%–100% B, 24–30 min: 100% B, 30–34 min: 100%–0.5% B, and 34–39 min: 0.5% B; volume of extract injected: 10 µL. Amounts of compounds were calculated on the basis of the peak area relative to the internal standard. The identification of phenolic acids and flavonoids was based on their retention times and specific UV spectra (if specific standards are commercially available), as well as mass spectrometry. The flavonoid and phenolic acid contents were presented in µmol g^−1^ DW.

### 2.6 Determination of Nitrate Contents

The nitrate content was determined using the Reflectoquant nitrate test based on the principle of reflectometry (remission photometry). In brief, 50 mg of dried and pulverized vegetable samples (*n* = 3 for each plant treatment) were diluted in 10 mL of distilled water by mixing for 15 min using an electric stirrer (VARIOMAG Electronicrüher POLY 15) and cylindrical magnetic stirrer bars (PTFE 20 × 6 mm^2^) at a speed of 150 revolutions min^−1^. To obtain clear extracts, samples were sieved, and nitrate concentration was measured reflectometrically using Refloctoquant test strips (Stock code: 1.16995.0001; range: 3–90 mg L^−1^) on a RQflex 20 reflectometer (Merck KGaA, D‐64293 Darmstadt, Germany) calibrated using a recalibration set (Supelco 1.16954.0001; 84.6%). The results were expressed as mg kg^−1^ FW.

### 2.7 Statistical Data Analysis

To evaluate the effect of the solar drying treatments on the minerals, secondary plant metabolites, and nitrate content of the selected vegetables, means were calculated using Microsoft Excel, and the data obtained were subjected to a one‐way analysis of variance (ANOVA) using IBM SPSS Statistics 29.0. Tukey's HSD test was used to separate the means and statistical significance obtained at *p *≤ 0.05. Results were presented as mean ± standard deviation (SD) of triplicate analyses (*n* = 3).

## 3 Results

### 3.1 Drying Performance of the Passive Solar Dryers

**FIGURE 2:**
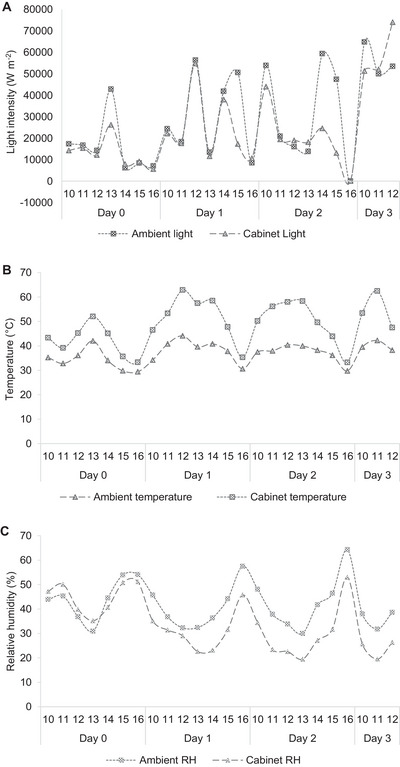
Variation in ambient and cabinet light intensity (A), temperature (B), and relative humidity (C) in passive direct solar dryer (PDSD) during the drying of Abyssinian mustard, pumpkin leaves, and amaranth. Time of the day is shown in 1‐h intervals between 900 and 1700 h.

**FIGURE 3:**
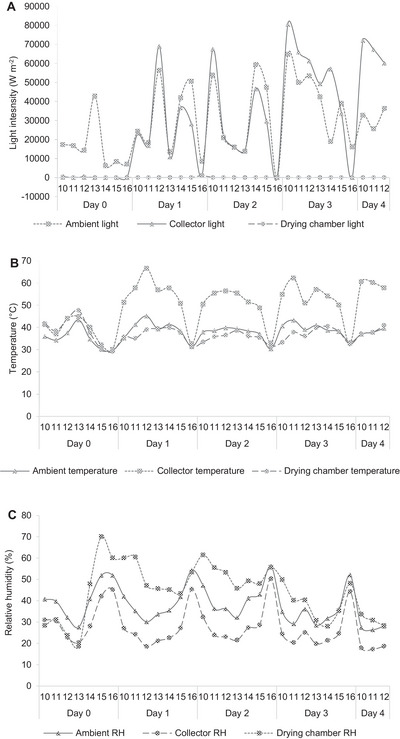
Variation in the solar radiation (A), temperature (B), and relative humidity (C) in the ambient, collector, and drying chamber of PISD during the drying of Abyssinian mustard, pumpkin leaves, and amaranth. Time of the day is shown in 1‐h intervals between 900 and 1700 h.

A total drying duration of 74 and 101 h was required to completely dry the vegetable samples using PDSD and PISD, respectively. The variations in the light intensity, temperature, and relative humidity using both PDSD and PISD are presented in Figures 2 and 3, respectively. For PDSD, the mean light intensity, temperature, and relative humidity in the ambient and drying cabinet conditions during the drying duration were 36,851 and 29,542 W m^−2^, 38°C and 52°C, and 43% and 36%, respectively. For PISD, the mean light intensity, temperature, and relative humidity in the ambient, collector, and drying chamber during the drying duration were 29,554, 29,997, and 65 W m^−2^; 38°C, 49°C, and 35°C; and 38%, 27%, and 44%, respectively. Following solar drying, the moisture contents in amaranth, Abyssinian mustard, and pumpkin leaves were lowered from initial values of 89%, 91%, and 86%, respectively, to final values of 5%, 6%, and 6%, respectively (PDSD), and to 6%, 7%, and 9%, respectively (PISD).

### 3.2 Effect of Solar Drying and Storage Treatments on the Carotenoid Contents of AIVs

The carotenoid contents in the selected AIVs following the different drying treatments are indicated in Table 2. Four different carotenoids, namely, ß‐carotene, lutein, violaxanthin, and neoxanthin, were analyzed, with lutein forming the highest percentage of the total carotenoid contents. For amaranth, Abyssinian mustard, and pumpkin leaves, the total carotenoid content ranged from 0.70 to 0.75, 0.54 to 0.61, and 0.93 to 1.13 mg g^−1^ DW, respectively (PDSD treatments), and from 0.76 to 0.85, 0.62 to 0.66, and 0.91 to 1.04 mg g^−1^ DW, respectively (PISD treatments) (Table 2).

**TABLE 2:** Chlorophyll and carotenoid contents of African indigenous vegetables (AIVs) following passive solar drying and storage.

Treatment	Chlorophylls			Carotenoids				
	Chlorophyll a (mg g^−1^ DW)	Chlorophyll b (mg g^−1^ DW)	Total chlorophyll (mg g^−1^ DW)	ß‐Carotene (mg g^−1^ DW)	Lutein (mg g^−1^ DW)	Violaxanthin (mg g^−1^ DW)	Neoxanthin (mg g^−1^ DW)	Total carotenoids (mg g^−1^ DW)
Amaranth								
Control	5.27 ± 0.50^a^	1.22 ± 0.12^a^	6.50 ± 0.62^a^	0.19 ± 0.03^bc^	0.40 ± 0.05^a^	0.06 ± 0.02^ab^	0.12 ± 0.03^a^	0.78 ± 0.14^a^
PDSD	5.68 ± 1.17^a^	1.42 ± 0.33^a^	7.10 ± 1.50^a^	0.12 ± 0.02^a^	0.41 ± 0.05^a^	0.04 ± 0.01^a^	0.12 ± 0.03^a^	0.70 ± 0.11^a^
PISD	5.58 ± 0.32^a^	1.42 ± 0.07^a^	7.00 ± 0.39^a^	0.24 ± 0.02^c^	0.42 ± 0.03^a^	0.08 ± 0.02^b^	0.12 ± 0.02^a^	0.85 ± 0.08^a^
PDSD‐S	5.87 ± 0.48^a^	1.43 ± 0.13^a^	7.30 ± 0.60^a^	0.15 ± 0.02^ab^	0.44 ± 0.03^a^	0.03 ± 0.00^a^	0.13 ± 0.02^a^	0.75 ± 0.04^a^
PISD‐S	4.78 ± 0.21^a^	1.21 ± 0.06^a^	5.99 ± 0.27^a^	0.22 ± 0.01^c^	0.38 ± 0.01^a^	0.06 ± 0.01^ab^	0.11 ± 0.02^a^	0.76 ± 0.05^a^
Abyssinian mustard								
Control	5.33 ± 0.20^b^	2.02 ± 0.08^c^	7.34 ± 0.28^b^	0.14 ± 0.03^b^	0.55 ± 0.06^b^	0.03 ± 0.00^d^	0.25 ± 0.05^b^	0.98 ± 0.14^b^
PDSD	2.93 ± 0.11^a^	1.16 ± 0.05^b^	4.09 ± 0.15^a^	0.06 ± 0.00^a^	0.33 ± 0.01^a^	0.01 ± 0.00^ab^	0.13 ± 0.01^a^	0.54 ± 0.02^a^
PISD	2.78 ± 0.54^a^	0.98 ± 0.17^ab^	3.76 ± 0.71^a^	0.15 ± 0.03^b^	0.34 ± 0.06^a^	0.03 ± 0.00^cd^	0.14 ± 0.03^a^	0.66 ± 0.09^a^
PDSD‐S	3.00 ± 0.25^a^	1.23 ± 0.05^b^	4.23 ± 0.30^a^	0.08 ± 0.02^a^	0.38 ± 0.04^a^	0.00 ± 0.00^a^	0.14 ± 0.02^a^	0.61 ± 0.07^a^
PISD‐S	2.48 ± 0.20^a^	0.87 ± 0.07^a^	3.35 ± 0.27^a^	0.16 ± 0.01^b^	0.32 ± 0.02^a^	0.02 ± 0.00^bc^	0.12 ± 0.02^a^	0.62 ± 0.04^a^
Pumpkin leaves								
Control	3.77 ± 0.13^a^	1.13 ± 0.28^a^	4.90 ± 0.15^a^	0.19 ± 0.02^c^	0.70 ± 0.03^c^	0.02 ± 0.01^a^	0.25 ± 0.02^b^	1.16 ± 0.09^b^
PDSD	6.14 ± 0.29^c^	2.23 ± 0.0^d^	8.37 ± 0.38^c^	0.15 ± 0.02^ab^	0.68 ± 0.05^bc^	0.05 ± 0.01^ab^	0.25 ± 0.01^b^	1.13 ± 0.09^b^
PISD	4.91 ± 0.40^b^	1.58 ± 0.09^bc^	6.49 ± 0.49^b^	0.22 ± 0.01^c^	0.59 ± 0.02^ab^	0.07 ± 0.01^b^	0.16 ± 0.03^a^	1.04 ± 0.02^ab^
PDSD‐S	4.98 ± 0.21^b^	1.86 ± 0.10^cd^	6.83 ± 0.30^b^	0.12 ± 0.01^a^	0.58 ± 0.03^a^	0.03 ± 0.01^a^	0.20 ± 0.01^ab^	0.93 ± 0.04^a^
PISD‐S	4.09 ± 0.30^a^	1.34 ± 0.08^ab^	5.42 ± 0.38^a^	0.18 ± 0.02^bc^	0.50 ± 0.05^a^	0.07 ± 0.02^b^	0.16 ± 0.01^a^	0.91 ± 0.09^a^

*Note*: PDSD‐S: 30‐day storage of passive direct solardried samples; PIDSD‐S: 30‐day storage of passive indirect solardried samples. Values are indicated as mean ± SD (*n* = 3). Different letters indicate significant differences between all variants (Tukey's HSD test, *p* ≤ 0.05).

Abbreviations: PDSD: passive direct solar dryer; PISD: passive indirect solar dryer.

For amaranth, there was no significant difference in the total carotenoid contents between the treatments and the control (Table 2). For Abyssinian mustard, however, a significant decline was observed in the total carotenoid contents in the treatments (45% PDSD, 33% PISD, 38% PDSD‐S, and 37% PISD‐S) compared to the control (Table 2). For pumpkin leaves, the total carotenoid contents were not significantly different between both PDSD and PISD and the control (Table 2). Although no statistically significant difference was observed in the total carotenoid content between PISD‐S and PISD for pumpkin leaves, a significant decrease (18%) of the total carotenoid content was observed in PDSD‐S compared to PDSD.

### 3.3 Effect of Solar Drying and Storage Treatments on the Chlorophyll Contents of AIVs

The chlorophyll contents in the selected AIVs following the different drying treatments are shown in Table 2. For amaranth, Abyssinian mustard, and pumpkin leaves, the total chlorophyll contents ranged from 7.10 to 7.30, 4.09 to 4.23, and 6.83 to 8.37 mg g^−1^ DW, respectively (PDSD treatments), and from 5.99 to 7.00, 3.35 to 3.76, and 5.42 to 6.49 mg g^−1^ DW, respectively (PISD treatments). For amaranth, the total chlorophyll contents were not statistically significant between all treatments. For Abyssinian mustard, a significant decline was revealed in the total chlorophyll contents in the treatments (44% PDSD, 42% PDSD‐S, 49% PISD, and 54% PISD‐S) compared to the control. However, no statistically significant difference was observed in the total chlorophyll contents for Abyssinian mustard between PDSD and PDSD‐S, and between PISD and PISD‐S. For pumpkin leaves, except in PISD‐S, a significantly higher total chlorophyll content (71% PDSD, 39% PDSD‐S, and 32% PISD) was observed in the treatments compared to the control. However, significant decreases were observed in the total chlorophyll contents for pumpkin leaves in PDSD‐S (18%) compared to PDSD and in PISD‐S (16%) compared to PISD.

### 3.4 Effect of Solar Drying and Storage Treatments on the Flavonoid Contents of AIVs

**FIGURE 4:**
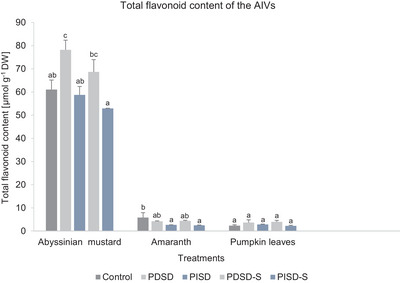
Effect of passive direct solar drying (PDSD) and passive indirect solar drying (PISD) followed by a 30‐day storage of passive direct solardried samples (PDSD‐S), and 30‐day storage of passive indirect solardried samples (PIDSD‐S) on the total flavonoid content of Abyssinian mustard, amaranth, and pumpkin leaves. Values are indicated as mean ± SD (*n* = 3). Different letters indicate significant differences between all variants (Tukey's HSD test, *p* ≤ 0.05). AIV, African indigenous vegetable; DW, dry weight.

The specific flavonoids detected in each AIV are indicated in Table S1, with higher quantities of quercetin rutinoside (amaranth and pumpkin leaves) and kaempferol dihexoside derivative (Abyssinian mustard) recorded. For amaranth, Abyssinian mustard, and pumpkin leaves, the total flavonoid content ranged from 4.17 to 4.38, 68.67 to 78.20, and 3.66 to 3.97 mg g^−1^ DW, respectively (PDSD treatments), and from 2.45 to 2.67, 52.90 to 58.73, and 2.23 to 2.83 mg g^−1^ DW, respectively (PISD treatments) (Figure 4).

For amaranth, there were no significant differences in the total flavonoid contents between the control and the treatments except for PISD and PISD‐S (Figure 4). A significant decrease was observed in the total flavonoid content in both PISD (54%) and PISD‐S (58%) compared to the control. For Abyssinian mustard, except for PDSD, the total flavonoid contents were not significantly different between the treatments and the control (Figure 4). Similarly, for pumpkin leaves, the total flavonoid content between the control and the treatments was not significantly different (Figure 4).

### 3.5 Effect of Solar Drying and Storage Treatments on the Phenolic Acid Contents of AIVs

**FIGURE 5:**
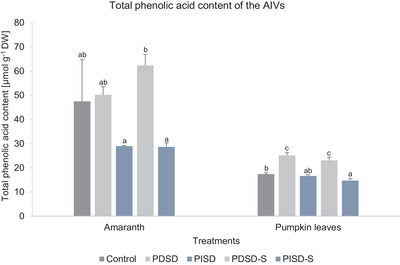
Effect of passive direct solar drying (PDSD), passive indirect solar drying (PISD), 30‐day storage of passive direct solardried samples (PDSD‐S), and 30‐day storage of passive indirect solardried samples (PIDSD‐S) on the total phenolic acid content of amaranth and pumpkin leaves. Values are indicated as mean ± SD (*n* = 3). Different letters indicate significant differences between all variants (Tukey's HSD test, *p* ≤ 0.05). AIV, African indigenous vegetables; DW, dry weight.

Five specific phenolic acids were detected in both amaranth and pumpkin leaves, with higher quantities of caffeoyl quinic acid and hydroxyferulyol derivate in both AIVs, respectively (Table S1). No specific phenolic acids were detected in Abyssinian mustard following the different treatments and therefore not accounted for in the present study. For amaranth and pumpkin leaves, the total phenolic acid contents ranged from 50.18 to 62.35 and 23.04 to 25.12 mg g^−1^ DW, respectively (PDSD treatments), and from 28.64 to 28.95 and 14.71 to 16.59 mg g^−1^ DW, respectively (PISD treatments) (Figure 5).

The variations in the total phenolic acid contents for amaranth between the treatments and the control, between PDSD and PDSD‐S, and between PISD and PISDS‐S, were not statistically significant (Figure 5). For pumpkin leaves, a significant increase was observed in the total phenolic acids content in both PDSD (45%) and PDSD‐S (33%) compared to the control (Figure 5). Compared to the control, the total phenolic acid content for pumpkin leaves was relatively maintained in PISD but significantly decreased in PISD‐S (15%).

### 3.6 Effect of Solar Drying and Storage Treatments on the Nitrate Contents of AIVs

**FIGURE 6:**
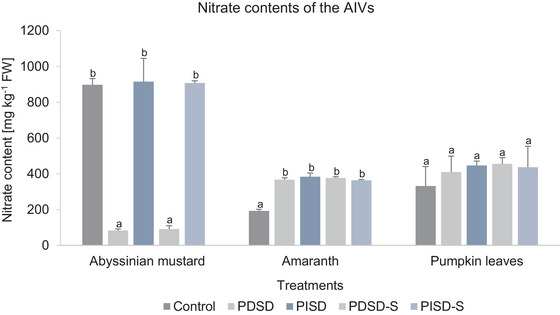
Effect of passive direct solar drying (PDSD), passive indirect solar drying (PISD), 30‐day storage of passive direct solardried samples (PDSD‐S), and 30‐day storage of passive indirect solardried samples (PIDSD‐S) on the nitrate content of Abyssinian mustard, amaranth, and pumpkin leaves. Values are indicated as mean ± SD (*n* = 3). Different letters indicate significant differences between all variants (Tukey's HSD test, *p* ≤ 0.05). AIV, African indigenous vegetables.

The mean nitrate content in amaranth, Abyssinian mustard, and pumpkin leaves as a result of the different solar drying treatments is presented in Figure 6.

Specifically, a significantly higher nitrate content was observed in amaranth (95%) in all the treatments compared to the control (Figure 6). The mean nitrate contents of amaranth in both solar drying treatments were not significantly different after 30 days of storage. For Abyssinian mustard, the nitrate content remained unaffected between the control and both PISD and PISD‐S samples (Figure 6). However, both PDSD and PDSD‐S samples revealed significantly lower nitrate contents compared to the control (Figure 6). For pumpkin leaves, no statistically significant differences between all treatments could be observed in the nitrate contents (Figure 6).

## 4 Discussion

### 4.1 Drying Performance of the Passive Solar Dryers

#### 4.1.1 Drying Duration

The drying performance of solar dryers evaluated through various parameters (e.g., drying duration, drying temperature, relative humidity, and moisture loss) is critical in vegetable dehydration as it impacts the quality of the dried products (Getahun et al. 2021). In this study, PDSD depicted a shorter drying duration, 27 h less than PISD. The difference in drying duration could be attributed to the higher temperature differences between the ambient and drying sections of the solar dryers, which serve as the main driving force for the drying process. According to Deng et al. (2021), compared to the other drying parameters, the quality of a dried product is mainly influenced by temperature. Babu et al. (2018) indicated that direct exposure of vegetable leaves to sunlight in PDSD generates a hot and dry microclimatic condition, facilitating a faster evaporation of moisture compared to PISD. In addition, despite possessing a black surface that is expected to have a higher solar energy absorption, PISD requires a longer time to warm up compared to PDSD, which is covered by a translucent plastic sheet. Cesar et al. (2019) also observed a higher difference (8.6 h) in the drying time between PDSD and PISD. The difference was attributed to possession of a higher solar irradiance collection area by the PDSD, which gives rise to higher solar radiation of the drying product, enabling a shorter drying duration as a result of increased drying temperatures. This also applies to the present study whereby, despite being smaller in size, PDSD depicted a higher solar irradiance collection area compared to PISD due to the possession of several surfaces that allow direct solar radiation of the leaves.

#### 4.1.2 Light Intensity

Throughout the drying duration, although a higher light intensity was recorded in the ambient than in the drying cabinet of the PDSD (Figure 2A), a higher light intensity was recorded in the collector compared to the ambient and the drying chamber of the PISD (Figure 3A). Light intensity in the collector (PISD) fluctuated corresponding to the ambient conditions, except on the first day, where the light intensity received in the collector was lower than the ambient condition. This could be due to the cloud cover experienced during the first day, as well as the fact that PISD requires a longer time to warm compared to PDSD due to the heavy metal material used for its construction. However, light intensity received in the drying chamber of the PISD remained relatively low compared to the ambient and collector conditions throughout the drying duration. Mohana et al. (2020) state that during solar drying, only a fraction of the incident solar radiation is absorbed and converted to thermal energy, which controls temperature changes and moisture removal. Practically, utilization of solar dryers is most suitable in regions that experience numerous and longer periods of sunshine (Rezaei et al. 2021). Tanzania, for example, collects an annual sunshine duration of 2800–3500 h and 4000–7000 W m^−2^ of solar radiation on a daily basis (Mongi and Ngoma 2022). Despite the abundance of the resource, with passive solar dryers, the respective solar energy generated can only be effectively utilized during daytime. According to Tiwari (2016), to reduce and control drying times, energy storage systems such as phase change materials that allow for continuous drying in the absence of sunshine should be incorporated in the design of passive solar dryers. However, inclusion of such systems into passive solar dryers would be impracticable for smallholder households due to their low per capita income, except strategies are developed for raising funds, for example, through farmers’ cooperative groups.

#### 4.1.3 Temperature, Relative Humidity, and Moisture Content

In the present study, higher temperatures were observed inside the drying cabinet of the PDSD in a pattern similar to the ambient temperature conditions (Figure 2B), attributed to the higher ambient solar radiation received. To the contrary, lower temperatures were recorded inside the drying chamber of the PISD than in the ambient conditions, in a pattern that varied with respect to the collector temperature, which was higher throughout the drying duration (Figure 3B). Castillo‐Téllez et al. (2024) recorded a mean temperature of 62°C in the collector of a PISD, with a lower mean value of 50°C in the drying chamber, contrasting with values obtained in the present study (collector temperature: 49°C, drying chamber temperature: 35°C). The decrease in temperature could be due to losses of heat experienced in the drying chamber from the inflowing hot air received from the collector (Castillo‐Téllez et al. 2024). Although the relative humidity inside the drying cabinet was relatively lower than the ambient conditions throughout the drying duration for PDSD (Figure 2C), the relative humidity inside the drying chamber of the PISD was generally higher compared to the ambient and collector values but gradually declined from the initial to the final days of drying (Figure 3C). The final moisture contents of the dried amaranth (5% PDSD, 6% PISD), Abyssinian mustard (6% PDSD, 7% PISD), and pumpkin leaves (6% PDSD, 9% PISD) were within the recommended values (<10%) for dried leafy vegetables (Ayua et al. 2016). Apart from the effect of the weather and drying conditions (Bayang et al. 2024), variations in the final moisture contents among the three AIVs could be mainly due to varietal differences. Different vegetables have different rates of water loss from their surfaces depending on the leaf size and shape, surface area, and the number and size of pore spaces. During the drying of leafy vegetables, a control of both temperature and moisture content is critical (Babu et al. 2018). Moisture content determines the capacity for nutrient preservation and quality retention in dried products (Manchali et al. 2021). Fresh leafy vegetables are highly perishable and prone to microbial infestation and spoilage due to possession of higher moisture content (>70%), thus limiting their storability (Nyambaka et al. 2012; Nambafu et al. 2021). Therefore, the reduction of moisture content in AIVs is critical for the minimization of inherent enzyme and microbial activity, thus improving shelf life (Ndukwu et al. 2023).

### 4.2 The Impact of Passive Solar Drying and Storage Treatments on the Quality of AIVs

#### 4.2.1 Carotenoid Content

Drying of fresh vegetable leaves using PDSD and PISD resulted in an acceleration of the synthesis or a retention of the total carotenoid content in the selected AIVs except in Abyssinian mustard, where a significant decrease was observed compared to the control (oven dried). A higher retention of the total carotenoid content was observed in PISD compared to PDSD in amaranth (100%) and Abyssinian mustard (67%). On the other hand, PDSD resulted in a higher retention of the total carotenoids content (97%) in pumpkin leaves compared to PISD (90%). Although the variation in the total carotenoid content retention in the AIVs could be due to species and varietal differences, the dryer type and resulting drying conditions also had an effect. Contrasting to the present study, a study by Maiman et al. (2021) found significantly higher carotenoid retention in tomatoes following solar drying (63.7 mg g^−1^ DW) and storage for 3–6 months (88.6–88.9 mg g^−1^ DW) compared to the fresh samples (41.9 mg g^−1^ DW). The variations were associated with low temperature (45°C) and short drying duration (24 h). Studies have indicated that the duration of the drying time exerts a strong influence on the retention of carotenoids compared to the drying temperature (Muratore et al. 2008; Lau et al. 2018). This was, however, inconsistent with the present study whereby longer drying duration did not significantly affect the carotenoid contents in PISD compared to PDSD samples. A study by Oliveira et al. (2016) associated losses in carotenoid contents (34%–92%) to longer drying time and longer exposure to harmful ultraviolet light following solar drying of vegetables. Although drying methods that prevent direct irradiation of the dried product could minimize the losses, they result in longer drying durations, which could require further optimizations, especially when drying products with higher moisture contents and thick cuticles (Oliveira et al. 2016). The significantly higher losses in carotenoids observed in Abyssinian mustard could be attributed to possession of a thicker cuticle compared to amaranth and pumpkin leaves, possibly resulting in a retardation of quick drying processes and thus followed by undesired biochemical processes, for example, carotenoid degradation. A 30‐day storage of the PDSD samples resulted in an enhanced retention of the total carotenoid content in the selected AIVs except for pumpkin leaves, where a significant decline (20%) was observed compared to the initial value. Storage of PISD samples resulted in a decline, though not significant, in the total carotenoid content of the AIVs, equivalent to a retention of 89% (amaranth), 94% (Abyssinian mustard), and 87% (pumpkin leaves). Literature indicates that the degradation of carotenoids in perishable products such as vegetables occurs due to oxidative reactions catalyzed by the enzyme lipoxygenase (Sturm and Hensel 2017; Onwude et al. 2021). Moreover, carotenoid breakdown could take place via isomerization reactions where carotenoid molecules are converted into different isomers, depending on the drying product characteristics and the drying conditions, mainly temperature (Onwude et al. 2021). The present findings indicate that passive solar drying and storage lead to quality preservation of AIVs in terms of total carotenoid retention, depending not only on the drying conditions but also on the vegetable species.

#### 4.2.2 Chlorophyll Content

Drying fresh vegetables using PDSD and PISD resulted in an accelerated synthesis or retention of the total chlorophyll content in amaranth and pumpkin leaves, but not in Abyssinian mustard, where a significant decline was observed compared to the control. Specifically, fresh vegetable samples dried using PDSD led to a substantial retention of the total chlorophyll content in both amaranth and pumpkin leaves (100%) compared to the control. However, only a retention of 56% (PDSD) and 51% (PISD) was observed in dried Abyssinian mustard samples compared to the control. Previous studies have associated higher drying temperatures (60–100°C) with higher degradation of total chlorophyll contents in leafy vegetables (Cui et al. 2004; Mashitoa et al. 2021). The drying temperatures recorded for the two solar dryers in the present study (PDSD: 52°C, PISD: 35°C) were lower compared to the values of the above‐mentioned studies. With the exception of Abyssinian mustard, the temperature range (35–52°C) could be the possible explanation for the high contents of the total chlorophylls observed in the AIVs in the present study. Moreover, a plausible cause of the outcome could be the differences in the drying duration and uniformity of air flow within the drying trays of the two solar dryers (Plabon et al. 2024). According to Pardeshi et al. (2013) and Mehta et al. (2017), a faster drying rate results in a shorter drying duration, allowing only for a reduced degradation effect on the chlorophyll pigments and thus a higher retention. This could be the reason for higher retentions of the total chlorophyll content observed in PDSD compared to PISD treatments. The total chlorophyll content was maintained in both amaranth and Abyssinian mustard (100%) following a 30‐day storage of PDSD samples. However, a significant decline of the total chlorophyll content was observed in PDSD pumpkin leaves (18%) following the 30‐day storage duration. To the contrary, storage of PISD samples experienced a decline in the total chlorophyll content (amaranth: 14%, Abyssinian mustard: 11%), significantly higher in pumpkin leaves (16%). Similar to the present findings, Mehta et al. (2017) observed a higher retention of the chlorophyll content in capsicum and bitter gourd following PDSD. According to Sturm and Hensel (2017), chlorophyll degradation during the processing and storage of products is initiated through tissue breakdown linked to sensitivity to several factors, including light, temperature, time, oxygen, enzymes, and pH. The mechanism of degradation of chlorophylls has been described as occurring at higher temperatures through their conversion to pheophytins (pheophorbide‐phytylesters), which are Mg‐free components of chlorophyll, or through chlorophyllase and lipoxidase enzymatic‐based reactions (Prinsi et al. 2019; Chaves et al. 2022). Although these findings imply that passive solar drying and storage are effective in quality preservation of AIVs in terms of chlorophyll retention, the drying temperature, drying duration, and vegetable type and species are important influencing factors.

#### 4.2.3 Flavonoid Content

Drying of fresh vegetable leaves using PDSD and PISD maintained the total flavonoid contents, except for amaranth samples (PISD), where a significant decline was found. However, specifically, irrespective of the vegetable species, PDSD resulted in higher total flavonoid contents (amaranth: 72%, Abyssinian mustard and pumpkin leaves: 100%), compared to PISD (amaranth: 47%, Abyssinian mustard: 96%, pumpkin leaves: 100%). Nevertheless, the present findings imply that both PDSD and PISD are effective in quality preservation in terms of the higher retention (>47%) of the total flavonoid content. Presumably, a possible reason for the outcome could be mainly the differences in the type and design of the solar dryers, which, as described earlier, influence the key drying parameters such as temperature, relative humidity, and duration. In the present study, PDSD resulted in a relatively shorter drying time compared to PISD, leading to a reduced exposure of the drying leaves to further degradation. According to Vidinamo et al. (2022), the variation in the total flavonoid content could be attributed to the influence of biochemical changes that occur due to drying vegetables at temperatures of 40–80°C. For example, higher extractability from respective samples as a result of the breakdown of leaf components (e.g., cell wall) and associated release from sequestration could result in increased total flavonoid content post‐drying (Capanoglu 2014; Vidinamo et al. 2022). In the present study, such a scenario could be applicable to Abyssinian mustard (PDSD) and pumpkin leaves (PDSD, PISD), which had increased total flavonoid content compared to the control. Further, the interaction between enzymatic and non‐enzymatic reactions, such as the effect due to peroxidase, polyphenol oxidase, and glycosidase enzymes, has been described as a possible contributor to flavonoid degradation during postharvest (Terefe et al. 2009; Plabon et al. 2024). Relatedly, Gunathilake et al. (2018) argue that the forms of the single flavonoids found in a particular plant matrix and the nature of the leaves (vegetable species) determine the variations in the total flavonoid content following processing. A 30‐day storage of the vegetables after respective solar drying treatments did not result in a significant decline of the total flavonoid contents. In particular, the storage of PDSD samples resulted in a higher retention of the total flavonoid contents (100%) for all three AIVs, as compared to the storage of PISD samples (amaranth: 93%, Abyssinian mustard: 90%, pumpkin leaves: 79%). The non‐significant variations in the total flavonoid content suggest that storage of solar‐dried vegetables preserves quality in terms of total flavonoid content retention for a maximum of 30 days of storage.

#### 4.2.4 Phenolic Acid Content

In this study, the use of PDSD resulted in a significantly higher content (amaranth and pumpkin leaves: 100%) of the total phenolic acids compared to PISD (amaranth: 61%, pumpkin leaves: 85%). Kirakou et al. (2017) obtained similar results, where a higher retention (100%) was observed in solardried vegetables. The results suggest that both PDSD and PISD result in quality preservation of AIVs in terms of the retention of total phenolic acids. However, as shown by the present findings, the effectiveness of the preservation of the total phenolic acids depends on the type of drying technology and associated drying mechanism. The increase in total phenolic acid content in PDSD samples is attributable to the effect of higher temperatures, possibly resulting in heat‐induced biochemical reactions (Netshimbupfe et al. 2023). Previous studies have shown that phenolic compounds are accumulated and bound in plant cellular structures (e.g., proteins and polysaccharides) and that high temperatures increase their extractability and availability due to the breakdown of covalent bonds and cellular components (Udomkun et al. 2020; Tan et al. 2023). In addition, higher temperatures have been stated as increasing the inactivation of enzymes responsible for the oxidation of phenolic acids (polyphenol oxidases and peroxidases) (Li et al. 2018; Ng et al. 2020), thus preventing further degradation of phenolic acids through improved stability (Simões et al. 2015; Moyo et al. 2018). The lower total phenolic acid content obtained in PISD could be attributed to extended drying times, which have been stated as resulting in higher losses of phenolic compounds due to an accelerated enzyme activity (Kerkhofs et al. 2005; Mbondo et al. 2018). The total phenolic acid content in both amaranth and pumpkin leaves was maintained following a 30‐day storage duration. Compared to the initial total phenolic acid content of the PDSD samples, storage resulted in a retention of 100% (amaranth) and 92% (pumpkin leaves) of the total phenolic acid content. Similarly, a retention of 99% (amaranth) and 89% (pumpkin leaves) of the total phenolic acid content was found in PISD samples following a 30‐day storage duration. This implies that ambient temperature storage of dried AIVs for a maximum of 1 month would lead to an insignificant degradation of total phenolic acids.

#### 4.2.5 Nitrate Content

In general, in the present study, the nitrate content in passive solardried AIVs was found to be higher than the values in the control (ovendried). Apart from the drying method applied, the differences in the nitrate contents in the solardried samples observed could be attributed mainly to the genotypic differences among the AIVs (Bahadoran et al. 2016), as illustrated by the different initial nitrate contents in the control (Figure 6). This is mainly due to the fact that different plants have different nitrate absorption capacities (Qasemi et al. 2024). According to Gottardi et al. (2012), factors such as light intensity and temperature could contribute to plant stress, resulting in increased accumulation of nitrate content. During growth, previous studies have associated nitrate accumulation in leafy vegetable tissues with low light intensity, whose accumulation results in restricted activity of the enzyme nitrate reductase (Guadagnin et al. 2005; Mola et al. 2019). The activity status of the enzyme nitrate reductase could also be linked to the variations in the nitrate levels in AIVs after solar drying, although reports describing the exact mechanisms during solar drying could not be found in literature. Moreover, in the present study, a 30‐day storage of dried vegetable samples did not result in significant changes in the nitrate content compared to the initial dried vegetable samples of all three AIVs. This implies that only negligible changes would occur in the nitrate content of solardried vegetables, at least over a 30‐day storage period. Previous studies have, however, associated ambient temperature storage with significant decreases in the nitrate content of vegetables (Chung et al. 2004; Hmelak Gorenjak and Cencič 2012, probably due to unrestricted endogenous nitrate reductase activity (Chung et al. 2005). The nitrate contents obtained following the passive solar drying treatments (PDSD: 80–460 mg kg^−1^ FW, PISD: 360–910 kg^−1^ FW) were below the limits stipulated by the European Commission Regulation (EC No. 915/2023) for fresh leafy vegetables (2000–4500 mg kg^−1^ FW) (Enri et al. 2024). This implies that consumption of the solardried AIVs is considered nutritionally safe in terms of nitrate levels. Despite this, important considerations for the reduction of nitrate concentration in leafy vegetables for all key AIV value chain actors include the adoption of good agricultural practices (Parks et al. 2012) and the enactment and enforcement of regulations regarding the allowable limits (León and Luzardo 2020).

## 5 Conclusion

The present study demonstrated that both PDSD and PISD are efficient in the preservation of AIVs, in terms of secondary plant metabolite contents. However, PDSD was more efficient compared to PISD due to the utilization of a shorter drying time. Except for the total flavonoid and total chlorophyll contents, there were no significant variations in the quality aspects following ambient storage of solardried AIVs for 30 days. These findings suggest that passive solar drying is effective in the preservation of secondary plant metabolite contents (i.e., carotenoids, chlorophylls, flavonoids, and phenolic acids) of AIVs for at least 30 days of ambient temperature storage. Due to the limitations of previous studies, which are mainly on pre‐treatments, conducted under controlled conditions, or based on active or mixed‐mode solar dryers, further empirical studies on the effects of passive solar dryers on AIVs are necessary. Future studies should focus on the sensory and consumer acceptance evaluations of passive solardried AIVs as well as their microbial quality, which are important aspects in food quality and food safety. Notwithstanding, due to its effectiveness in quality preservation and thus potential for the alleviation of PHLs, it is recommended that passive solar dryers be promoted for ease of adoption and utilization among smallholder farmers and households. Particularly, the PDSD should be given strong consideration due to its demonstrated effectiveness and ease and simplicity of construction using locally available resources. This could be facilitated by conducting hands‐on knowledge dissemination seminars and workshops on the design and construction of passive solar dryers with cheap and locally accessible materials. For optimized drying performance of the PDSD, cover sheets with capacities to screen harmful light rays, as well as naturally operated fans (e.g., wind, gravity), should be incorporated in future designs. Future studies on how to overcome limitations posed by adverse weather conditions (e.g., rainfall) on passive solar drying are also recommended.

## Author Contributions

**James S. Chacha**: conceptualization, investigation, formal analysis, methodology, data curation, validation, visualization, writing – original draft, writing – review and editing. **Nadja Förster**: methodology, data curation, validation, review and editing, supervision. **Susanne Huyskens‐Keil**: methodology, investigation, review and editing, supervision. **Christian Ulrichs**: resources, project administration, funding acquisition, supervision. **Constance Rybak**: conceptualization, review and editing, project administration, funding acquisition, supervision.

## Ethics Statement

The authors have nothing to report.

## Conflicts of Interest

The authors declare no conflicts of interest.

## Supporting information




**Supplementary Table**: jfds70800‐sup‐0001‐tableS1.docx

## Data Availability

Data will be made available on request.
