# Supplementary material for: Impact of Passive Solar Drying and Storage on Secondary Plant Metabolites and Nitrate Contents of Abyssinian Mustard, Amaranth, and Pumpkin Leaves
Source: J Food Sci. 2026 Jan 7;91(1):e70800. doi: 10.1111/1750-3841.70800 (PMC12780662; doi:10.1111/1750-3841.70800)
Supplement: Supplementary file 1 — Supplementary Table: jfds70800‐sup‐0001‐tableS1.docx [file JFDS-91-0-s001.docx]

Supplementary Table 1A: Specific flavonoids & phenolic acids of amaranth

| Amaranth | hydroxyferuloyl derivate [µmol/g DW] | feruloyl derivate [µmol/g DW] | caffeoyl quinic acid [µmol/g DW] | coumaryl quinic acid [µmol / g DW] | feruloyl quinic acid [µmol / g DW] | Total phenolic acids [µmol/g DW] | quercetin rutinoside [µmol / g DW] | Kaempferol glucoside [µmol / g DW] | Total flavonoids [µmol/g DW] |
| --- | --- | --- | --- | --- | --- | --- | --- | --- | --- |
| Control | 8.03 ± 2.89^ab^ | 5.38 ± 2.07^b^ | 22.89 ± 8.19^ab^ | 0.85 ± 0.30^a^ | 10.33 ± 3.76^a^ | 47.48 ± 17.20^ab^ | 5.15 ± 1.94^b^ | 0.68 ± 0.18^b^ | 5.83 ± 2.12^b^ |
| PDSD | 7.45 ± 0.42^ab^ | 3.93 ± 0.14^ab^ | 28.71 ± 2.36^bc^ | 1.10 ± 0.03^a^ | 8.99 ± 0.57^a^ | 50.18 ± 3.36^ab^ | 3.58 ± 0.21^ab^ | 0.58 ± 0.10^ab^ | 4.17 ± 0.23^ab^ |
| PISD | 4.39 ± 0.06^a^ | 3.20 ± 0.21^ab^ | 13.98 ± 0.22^a^ | 1.07 ± 0.02^a^ | 6.30 ± 0.09^a^ | 28.95 ± 0.30^a^ | 2.30 ± 0.07^a^ | 0.35 ± 0.01^a^ | 2.66 ± 0.06^a^ |
| PDSD-S | 9.16 ± 0.83^b^ | 4.07 ± 0.22^ab^ | 37.51 ± 2.95^c^ | 1.27 ± 0.08^a^ | 10.33 ± 0.54^a^ | 62.35 ± 4.55^b^ | 3.79 ± 0.29^ab^ | 0.59 ± 0.03^ab^ | 4.38 ± 0.31^ab^ |
| PISD-S | 4.46 ± 0.24^a^ | 2.65 ± 0.57a | 14.42 ± 0.69^a^ | 0.95 ± 0.28^a^ | 6.17 ± 0.38^a^ | 28.64 ± 1.65^a^ | 2.13 ± 0.10^a^ | 0.33 ± 0.05^a^ | 2.45 ± 0.15^a^ |

Supplementary Table 1B: Specific flavonoids & phenolic acids of pumpkin leaves

| Pumpkin leaves | hydroxyferulyol derivate [µmol / g DW] | feruloyl derivate [µmol / g DW] | caffeoyl quinic acid [µmol / g DW] | coumaryl quinic acid [µmol / g DW] | feruloyl quinic acid [µmol / g DW] | Total phenolic acids [µmol/g DW] | quercetin rutinoside [µmol / g DW] | quercetin hexoside [µmol / g DW] | Kaempferol glucoside [µmol / g DW] | Total flavonoids [µmol/g DW] |
| --- | --- | --- | --- | --- | --- | --- | --- | --- | --- | --- |
| Control | 13.15 ± 0.16^b^ | 2.47 ± 0.39^a^ | 0.80 ± 0.15^a^ | 0.72 ± 0.07^a^ | 0.24 ± 0.06^a^ | 17.38 ± 0.58^b^ | 1.14 ± 0.22^a^ | 0.12 ± 0.03^a^ | 1.04 ± 0.15^a^ | 2.30 ± 0.40^a^ |
| PDSD | 18.56 ± 1.07^d^ | 3.82 ± 0.02^bc^ | 1.05 ± 0.11^a^ | 1.31 ± 0.15^b^ | 0.38 ± 0.03^a^ | 25.12 ± 1.14^c^ | 1.93 ± 0.70^a^ | 0.23 ± 0.14^a^ | 1.49 ± 0.35^ab^ | 3.66 ± 1.18^a^ |
| PISD | 11.21 ± 0.29^a^ | 2.96 ± 0.09^ab^ | 1.10 ± 0.47^a^ | 0.73 ± 0.12^a^ | 0.60 ± 0.06^b^ | 16.59 ± 0.55^ab^ | 1.47 ± 0.08^a^ | 0.16 ± 0.01^a^ | 1.20 ± 0.09^ab^ | 2.83 ± 0.18^a^ |
| PDSD-S | 16.50 ± 0.90^c^ | 3.91 ± 0.63^c^ | 1.03 ± 0.13^a^ | 1.26 ± 0.19^b^ | 0.33 ± 0.06^a^ | 23.04 ± 1.31^c^ | 1.99 ± 0.33^a^ | 0.23 ± 0.05^a^ | 1.75 ± 0.30^b^ | 3.97 ± 0.67^a^ |
| PISD-S | 10.24 ± 0.73^a^ | 2.67 ± 0.17^a^ | 0.74 ± 0.07^a^ | 0.49 ± 0.08^a^ | 0.57 ± 0.06^b^ | 14.71 ± 0.84^a^ | 1.17 ± 0.19^a^ | 0.12 ± 0.01^a^ | 0.94 ± 0.11^a^ | 2.23 ± 0.30^a^ |

Supplementary Table 1C: Specific flavonoids of Abyssinian mustard

| Abyssinian mustard | kaempferol dihexoside derivate [µmol / g DW] | kaempferol dihexoside-hydroxyferuloyl hexoside [µmol / g DW] | kaempferol dihexoside caffeoyl hexoside [µmol / g DW] | kaempferol trihexoside sinapoyl hexoside [µmol / g DW] | kaempferol dihexoside sinapoyl hexoside [µmol / g DW] | kaempferol trihexoside feruloyl hexoside [µmol / g DW] | kaempferol dihexoside feruloyl hexoside [µmol / g DW] | kaempferol dihexoside caffeoyl rhamnoside [µmol / g DW] | kaempferol dihexoside hydroxyferuloyl [µmol / g DW] | kaempferol dihexoside caffeoyl [µmol / g DW] | kaempferol dihexoside feruloylderivate [µmol / g DW] | kaempferol trihexoside disinapoyl hexoside [µmol / g DW] | kaempferol dihexoside rhamnoside [µmol / g DW] | kaempferol hexoside [µmol / g DW] | Total flavonoids [µmol/g DW] |
| --- | --- | --- | --- | --- | --- | --- | --- | --- | --- | --- | --- | --- | --- | --- | --- |
| Control | 10.57 ± 0.65^a^ | 10.21 ± 0.69^b^ | 7.90 ± 0.51^bc^ | 2.57 ± 0.16^ab^ | 8.86 ± 0.61^a^ | 2.72 ± 0.18^bc^ | 9.12 ± 0.59^ab^ | 2.90 ± 0.19^a^ | 0.79 ± 0.06^b^ | 0.77 ± 0.05^b^ | 1.54 ± 0.21^b^ | 2.33 ± 0.21^ab^ | 0.44 ± 0.04^abc^ | 0.31 ± 0.03^a^ | 61.03 ± 4.14^ab^ |
| PDSD | 15.61 ± 0.58^b^ | 11.85 ± 0.85^c^ | 8.73 ± 0.46^c^ | 3.05 ± 0.20^c^ | 15.61 ± 0.61^b^ | 3.72 ± 0.17^d^ | 12.18 ± 1.04^c^ | 2.92 ± 0.21^a^ | 0.29 ± 0.01^a^ | 0.14 ± 0.01^a^ | 0.88 ± 0.03^a^ | 2.60 ± 0.12^b^ | 0.38 ± 0.04^ab^ | 0.24 ± 0.01^a^ | 78.20 ± 4.17^c^ |
| PISD | 11.18 ± 0.78^a^ | 8.12 ± 0.51^a^ | 7.31 ± 0.49^ab^ | 2.61 ± 0.17^ab^ | 8.65 ± 0.66^a^ | 2.37 ± 0.15^ab^ | 8.86 ± 0.55^a^ | 2.93 ± 0.13^a^ | 0.90 ± 0.06^b^ | 0.93 ± 0.07^c^ | 2.04 ± 0.11^b^ | 1.95 ± 0.13^a^ | 0.57 ± 0.06^bc^ | 0.29 ± 0.03^a^ | 58.73 ± 3.64^ab^ |
| PDSD-S | 13.50 ± 1.38^b^ | 10.66 ± 0.50^bc^ | 7.91 ± 0.57^bc^ | 2.79 ± 0.09^bc^ | 13.71 ± 1.33^b^ | 3.11 ± 0.19^c^ | 10.71 ± 0.75^bc^ | 2.68 ± 0.19^a^ | 0.25 ± 0.03^a^ | 0.10 ± 0.02^a^ | 0.60 ± 0.31^a^ | 2.20 ± 0.25^ab^ | 0.27 ± 0.14^a^ | 0.19 ± 0.06^a^ | 68.67 ± 5.34^bc^ |
| PISD-S | 10.30 ± 0.55^a^ | 7.13 ± 0.13^a^ | 6.38 ± 0.02^a^ | 2.33 ± 0.03^a^ | 7.85 ± 0.03^a^ | 2.03 ± 0.02^a^ | 7.67 ± 0.03^a^ | 2.57 ± 0.02^a^ | 0.79 ± 0.02^b^ | 0.96 ± 0.02^c^ | 2.06 ± 0.31^b^ | 1.86 ± 0.12^a^ | 0.67 ± 0.16^c^ | 0.32 ± 0.10^a^ | 52.90 ± 0.09^a^ |
